# Avoiding unnecessary hospitalisation for patients with chronic conditions: a systematic review of implementation determinants for hospital avoidance programmes

**DOI:** 10.1186/s13012-020-01049-0

**Published:** 2020-10-21

**Authors:** Mitchell Sarkies, Janet C. Long, Chiara Pomare, Wendy Wu, Robyn Clay-Williams, Hoa Mi Nguyen, Emilie Francis-Auton, Johanna Westbrook, Jean-Frédéric Levesque, Diane E. Watson, Jeffrey Braithwaite

**Affiliations:** 1 Centre for Healthcare Resilience and Implementation Science, Australian Institute of Health Innovation, Macquarie University, New South Wales, Australia; 2 Centre for Health Systems and Safety Research, Australian Institute of Health Innovation, Macquarie University, New South Wales, Australia; 3 Agency for Clinical Innovation, New South Wales, Australia; 4 Centre for Primary Health Care and Equity, University of New South Wales, New South Wales, Australia; 5 Bureau of Health Information, New South Wales, Australia

**Keywords:** Chronic condition, Hospital avoidance, Value-based healthcare, Implementation science, Implementation determinants

## Abstract

**Background:**

Studies of clinical effectiveness have demonstrated the many benefits of programmes that avoid unnecessary hospitalisations. Therefore, it is imperative to examine the factors influencing implementation of these programmes to ensure these benefits are realised across different healthcare contexts and settings. Numerous factors may act as determinants of implementation success or failure (facilitators and barriers), by either obstructing or enabling changes in healthcare delivery. Understanding the relationships between these determinants is needed to design and tailor strategies that integrate effective programmes into routine practice. Our aims were to describe the implementation determinants for hospital avoidance programmes for people with chronic conditions and the relationships between these determinants.

**Methods:**

An electronic search of four databases was conducted from inception to October 2019, supplemented by snowballing for additional articles. Data were extracted using a structured data extraction tool and risk of bias assessed using the Hawker Tool. Thematic synthesis was undertaken to identify determinants of implementation success or failure for hospital avoidance programmes for people with chronic conditions, which were categorised according to the Consolidated Framework for Implementation Research (CFIR). The relationships between these determinants were also mapped.

**Results:**

The initial search returned 3537 articles after duplicates were removed. After title and abstract screening, 123 articles underwent full-text review. Thirteen articles (14 studies) met the inclusion criteria. Thematic synthesis yielded 23 determinants of implementation across the five CFIR domains. ‘Availability of resources’, ‘compatibility and fit’, and ‘engagement of interprofessional team’ emerged as the most prominent determinants across the included studies. The most interconnected implementation determinants were the ‘compatibility and fit’ of interventions and ‘leadership influence’ factors.

**Conclusions:**

Evidence is emerging for how chronic condition hospital avoidance programmes can be successfully implemented and scaled across different settings and contexts. This review provides a summary of key implementation determinants and their relationships. We propose a hypothesised causal loop diagram to represent the relationship between determinants within a complex adaptive system.

**Trial registration:**

PROSPERO 162812

Contributions to the literature • There is evidence of clinical effectiveness to support the use of hospital avoidance programmes for people with chronic conditions. Therefore, it is important to examine the factors associated with successful implementation to enable scaling of programmes across contexts and settings.

• We found that factors determining the implementation of chronic condition hospital avoidance programmes are related across all five domains of the Consolidated Framework for Implementation Research. A theorised causal loop diagram for the relationships between determinants is proposed.

• These findings progress the field of implementation science from classification to causality. Improvement strategies can be designed to target these related determinants.

## Background

Chronic conditions are the leading cause of death and disability [1] and pose a worldwide economic burden to health systems [2]. In 2019, almost one third of adults reported living with two or more chronic conditions within the Organisation for Economic Co-operation and Development (OECD) countries [2]. These conditions (e.g. chronic heart failure, chronic obstructive pulmonary disease) persist or reoccur over extended periods of time [3], accounting for high rates of repeated and potentially preventable hospitalisations with associated costs [4–6]. Increasing resources dedicated to management of chronic conditions in hospital settings is not deemed by governments and some commentators as sustainable [7–12]. To ensure appropriate care is delivered outside of inpatient settings, there has been a shift in policy and research towards value-based healthcare and reducing avoidable hospitalisations for chronic conditions [13, 14].

Value-based healthcare aims to maximise the benefits of care. Different schools of thought exist for how this can be best achieved; one is to maximise the health outcomes achieved per dollar spent [15, 16], another argues for ‘allocative value’ by distributing resources to ensure the right patient groups reach the right service at the right time [17]. In this review, we consider value from the perspective of achieving allocative value, which on a population level is thought to achieve ideal care at the lowest cost, without sacrificing quality and safety [13, 18]. One way to improve value for patients and minimise costs is to identify instances where hospitalisation may not be the most appropriate care. Initial presentations to hospital can be avoided or length of stay can be reduced, where appropriate care is delivered in other settings (e.g. subacute, ambulatory, or primary care) [19, 20]. Hospital avoidance programmes, such as multidisciplinary clinical management, patient education and self-management strategies, post-discharge planning and transitional care, hospital-at-home, and telemedicine, act to ensure the appropriate level of care is delivered in the right setting [21–23].

Multiple clinical effectiveness studies have established the benefits of hospital avoidance programmes for chronic conditions (e.g. reduced length of stay or readmission rates) [23, 24]. In light of these findings, there is an interest in implementing and scaling these benefits beyond their original contexts of application [14, 23]. Implementation science considers factors beyond, instead investigating the means by which effective interventions are translated into routine practice [25] and scaled to increase the coverage, range, and sustainability of services [26]. Implementation outcomes include the extent of an intervention’s adoption, penetration into the healthcare system, perceived acceptability and appropriateness among stakeholders, cost, and the fidelity to intended practice [27, 28]. Determinants of implementation success or failure are, therefore, any factors that influence these outcomes, including preparation for change, the capacity and nature of implementation, availability of resources, use of leverage, future sustainability, and trust between stakeholders [29, 30].

Specific to improving the management of chronic conditions, Kadu and Stolee [31] identified implementation determinants considered important to the environmental characteristics within organisations (e.g. inner setting organisational culture) and among individual healthcare professionals (e.g. attitudes and beliefs). Previous reviews that examine hospital avoidance programmes (not specific to chronic conditions) have been unable to consolidate barriers and facilitators to implementation due to the cohort-specific nature of primary studies reviewed [23]. An examination of implementation determinants for hospital avoidance programmes for people with chronic conditions is necessary to address this gap in the literature and inform the successful implementation and scaling of these programmes.

The primary aim of this review was to describe the implementation determinants for chronic condition hospital avoidance programmes. The secondary aim was to map the relationships between these determinants.

## Methods

### Search strategy

A systematic review (PROSPERO registration number 162812) was conducted in accordance with the Preferred Reporting Items for Systematic Reviews and Meta-analyses (PRISMA) statement [32]. Four databases (MEDLINE, PsycINFO, CINAHL, EMBASE) were searched in October 2019 with no date limits. The search strategy is presented in Additional File 1 and was limited to humans, the English language, and peer-reviewed publications only. Electronic database searches were supplemented by snowballing for additional articles via the reference list of identified systematic reviews relevant to the topic and included articles.

Reference details for all returned searches were downloaded into the electronic screening programme Rayyan [33], where three authors (MS, CP, WW) independently screened 5% (*n* = 177) of articles and tested these for inter-rater reliability. Inter-rater reliability was calculated by averaging Fleiss’ Kappa statistic for the three reviewers [34]. Although there was 97.2% agreement between the three reviewers, the generally high proportion of exclusion decisions and high rate of agreement between reviewers led to a Paradox 1 misrepresentation of the Kappa score (0.36) in this instance [35, 36]. Differing results were discussed and clarified before proceeding with the remaining title and abstract screening using one independent reviewer for each. Prior to full text screening, 5% of the remaining studies were independently screened by the three reviewers (MS, CP, WW) with no discrepancies identified. Full text review was then conducted for the remaining studies.

### Eligibility criteria

Articles that were included in the review addressed the implementation of hospital avoidance programmes for chronic care conditions. Studies were included if the research involved (1) a hospital avoidance programme, (2) targeted patients with a chronic condition, and (3) methods to identify implementation determinants. This third criterion was used to remove studies purely focussed on clinical effectiveness or utility, given our focus on factors influencing implementation outcomes. Hospital avoidance programmes had to align with the goal to reduce one or more of the following: emergency department (re)presentation, hospital (re)admission, length of stay, or unwarranted clinical variation [37]. Only six chronic condition groups were included: osteoarthritis, osteoporosis, renal disease, diabetes mellitus, chronic heart failure (CHF), and chronic obstructive pulmonary disease (COPD). These chronic conditions were chosen as they reflect the most common groups affecting hospital utilisation [38–42]. Both qualitative and quantitative studies were included. We excluded non-empirical studies, conference proceedings, and reviews.

### Data extraction and synthesis

Three reviewers (MS, CP, WW) independently piloted a structured data extraction tool on the same five articles, before extraction individually for the remaining articles. Any disagreements between reviewers were resolved via discussion. Extracted information included study details, design, setting, patient population, chronic condition, and details of the hospital avoidance programme. The thematic synthesis utilised both inductive and deductive methods described by Thomas and Harden [43], using NVivo [44]. This approach has three overlapping stages: (1) coding of text line-by-line, (2) development of descriptive themes, (3) generation of analytic themes.

#### Inductive and deductive coding

In stages 1 and 2, each of the included studies were inductively coded to identify determinants of implementation, building descriptive themes around how they affected the failure or success of programmes using the language and reasoning of the original paper’s authors. Coding was conducted by three reviewers (MS, WW, JCL). Methodological rigor was ensured through constant comparison, reflexive analysis, and peer debriefing. As each new study was coded, new codes were developed to capture the meaning and content of each sentence, leading to *n* = 44 total codes. Axial coding was performed during and upon completion to check consistency of interpretation and to build levels of coding, which resulted in a hierarchical tree structure. New codes were applied to groupings to produce descriptive themes.

At the end of stage 2, the descriptive themes represented implementation determinants presented in the language of the original primary studies. In stage 3, three reviewers (MS, JCL, RCW) interpreted how implementation determinants, captured in the descriptive themes, were related across the Consolidated Framework for Implementation Research (CFIR) domains [45]. This involved exploring new constructs (such as whether determinants were considered relevant to the inner setting or outer setting) that were not necessarily developed within studies but became apparent between studies. The process began by deductively allocating each of the descriptive themes to one of the five CFIR domains. These CFIR domains are the inner setting, outer setting, process, characteristics of the individuals, and characteristics of the interventions. We did not attempt to code the descriptive themes closely to the CFIR constructs, as stage 1 had been inductive. We then developed an analytical summary matrix by tabulating the domains and themes for each of the included studies within a table. From this, we identified determinants that were present across three or more of the included studies and occurred in combination with at least three other determinants. A fourth reviewer (WW) was consulted to discuss and develop final consensus. Disagreements were resolved by discussion and returning to the original open codes.

#### Identifying contingent and reciprocal relationships

Ideas about how these elements are connected emerged from the original data in the form of contingency relations (implication that changes in theme *b* is contingent upon theme *a*) and reciprocal relations (bidirectional forms of interaction between themes *a* and *b*) [46]. For example, in one study “contextual elements such as teamwork” was *contingent* upon the provision of support by “hospital leadership” when implementing COPD care bundles [47]. These relationships were coded by one researcher (MS) and were checked independently by another reviewer (WW), where no disagreements were identified. Once coded, the relationships between these determinants were mapped using the Vensim software (Ventana Systems, Inc) [48].

### Risk of bias assessment

Risk of bias was assessed using the validated Hawker Tool [49]. Each article was scored out of 36 across nine domains of study quality, which were then grouped according to the score into one of four categories: very poor, poor, fair, good. Three reviewers (MS, CP, WW) independently piloted the risk of bias tool on five of the included full text articles, with any discrepancies about using the tool discussed and resolved. The remaining articles were assessed independently against the tool. Any minor uncertainties were clarified as a group upon completion of the risk of bias assessment for the remaining included full texts. We did not report the differences in implementation determinants identified based on study quality, as determinants tend to reflect the focus of the original studies (e.g. patient factors, organisational factors) rather than methodological quality.

## Results

**Fig. 1:**
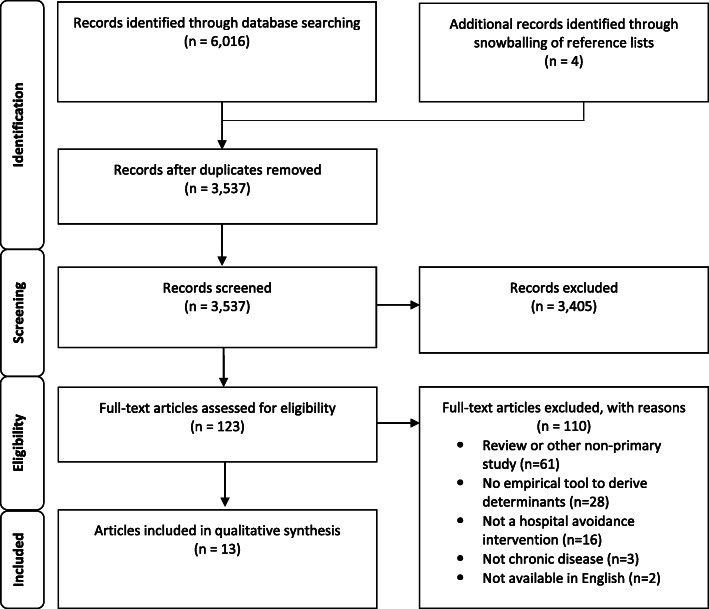
PRISMA flow diagram

The initial search returned 3537 articles after duplicates were removed. After title abstract screening, 123 articles were subjected to full-text review, of which 110 did not meet inclusion criteria. This resulted in 13 articles being included in this review (Fig. 1). Upon assessment, the quality of reporting was variable between studies. Quality assessment areas that were well reported included the abstract and title, introduction and aims, and implications and usefulness of the studies (Additional File 2). The main weaknesses were reporting of ethics and sources of bias and study transferability/generalisability.

Thirteen articles reported on fourteen hospital avoidance studies, using a variety of methodological approaches to examine their implementation. Five of these studies used a qualitative approach, four used primarily quantitative survey data, four used a mixed method approach, and the remaining study used a randomised controlled trial. Implementation determinants were generated from all study types in the thematic synthesis. Most of the studies came from the USA (*n* = 6), followed by Europe (*n* = 5), Canada (*n* = 1), and New Zealand (*n* = 1). The included studies focused on COPD (*n* = 6), heart failure (*n* = 5), and diabetes mellitus (*n* = 2), with one combined COPD and heart failure cohort. Further characteristics of included studies can be found in Table 1.

**Table 1:** Characteristics of included papers

Study	Country	Study type/design	Setting	Patient population	Sites	Chronic care condition	Type of hospital avoidance programme	Outcomes assessed
Acton et al. [50]	USA	Quantitative—RCT pilot	Hospital	Adult in-patients with type 1 or 2 DM, with home experience of using basal-bolus insulin	1	DM	Self-managed insulin	Length of stay
Axon et al. [51]	USA	Mixed methods	Various: hospital, home, hospices	CHF, COPD, pneumonia, AMI patients	68	CHF + COPD	1. Risk assessment 2. Education (teach back) 3. Follow-up (phone calls and appointments) 4. Transition (records, coaching), discharge summaries 5. Multidisciplinary rounds	Hospital readmissions
Benzo et al. [52]	USA	Qualitative	Hospital	Inpatient admitted with acute exacerbation of COPD	1	COPD	Pulmonary rehabilitation and exercise	Hospital readmissions
Fisher et al. [53]	USA	Qualitative	Hospital	Patients with severe exacerbations of COPD	7	COPD	Non-invasive ventilation	Length of stay
Hopkinson et al. [54]	UK	Mixed methods	Hospital	Inpatients admitted with acute exacerbation of COPD	1	COPD	1. Discharge care bundle 2. Post discharge follow-up phone call	Hospital readmissions
Lennox et al. [47]	UK	Qualitative	Hospital	Inpatients admitted with acute exacerbation of COPD	7	COPD	Care bundle	Hospital readmissions Length of stay
Morton et al. [55]	UK	Mixed methods	Hospital	Admitted patients with acute exacerbation of COPD	31	COPD	Admission and discharge care bundles	Hospital readmissions
Nguyen et al. [56]	Canada	Mixed methods	Hospital	HF patients (> 65) attending the general hospital Heart Function Clinic	1	CHF	Technology-based decision support to support self-care in older HF patients and their care partners	Hospital readmissions
Seys et al. [57]	Belgium, Italy, and Portugal	Quantitative	Hospital	Inpatients admitted with acute exacerbation of COPD	19	COPD	Care pathway	Hospital readmissions
Willemse et al. [58]	Belgium	Qualitative	Primary and secondary care	Community-based CHF patients	7	CHF	Telemonitoring and self-management	Hospital readmissions
Wood et al. [59] Study 1	USA	Quantitative	Hospital	Inpatients admitted with first diagnosis of HF in a military healthcare facility	1	CHF	Practice changes 1. Education tool which included instructions on medications, daily weights, exercise, sodium intake, reporting symptoms, recording follow-up appointments. 2. Making a patient follow-up appointment in HF facility within 10 days	Hospital readmissions
Wood et al. [59] Study 2	USA	Quantitative	Hospital	Patients with a history of HF discharged to participating SNFs in a civilian healthcare facility	1	CHF	Handoff protocol established to aid in the transition of care from inpatient to outpatient setting	Hospital readmissions
Wright et al. [60]	New Zealand	Quantitative–RCT	Hospital	Admitted with first diagnosis or an exacerbation of pre-existing HF	1	CHF	Self-management	Hospital readmissions
Yeager et al. [61]	USA	Qualitative	Hospitals and health centres	> 65 years diagnosed with DM plus one other chronic condition and Medicare eligible	6	DM	Care coordination model	Hospital admissions Emergency department presentations

*DM* diabetes mellitus, *CHF* congestive heart failure, *HF* heart failure, *AMI* acute myocardial infarction, *COPD* chronic obstructive pulmonary disease, *SNF* skilled nursing facility, *RCT* randomised controlled trial

### Descriptive themes: implementation determinants

**Fig. 2:**
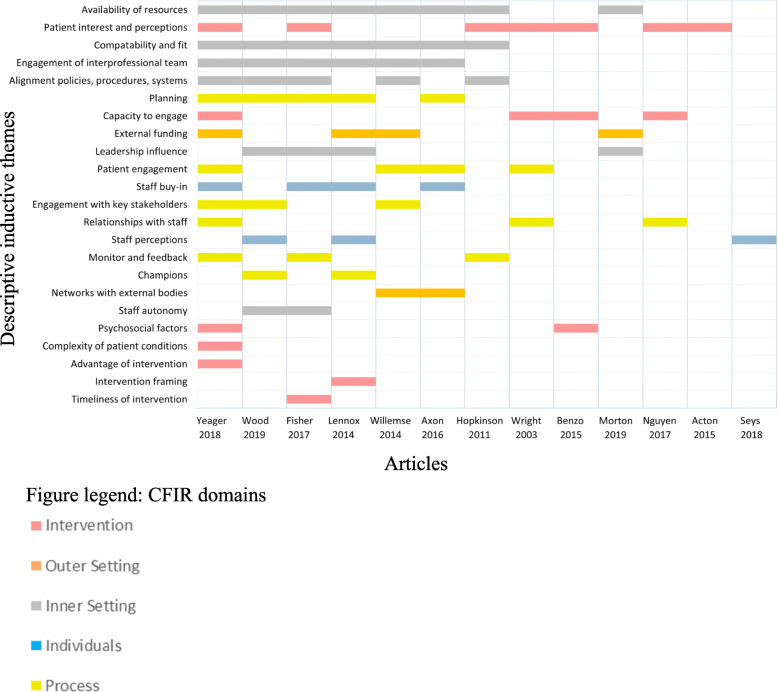
Reporting of implementation determinants in the included studies, across the Consolidated Framework for Implementation Research domains

The analysis yielded 23 implementation determinants (descriptive themes) across the five CFIR domains for chronic condition hospital avoidance programmes (Fig. 2). CFIR domains and categories, descriptive themes, and explanations, as well as exemplar quotes from the papers are provided in Table 2. Across the 13 articles, reporting on 14 studies, the key implementation determinants most frequently identified were the ‘availability of resources’ (*n* = 8 articles), ‘patient interest and perceptions’ (*n* = 7), ‘compatibility and fit of interventions’ (*n* = 7 articles), and ‘engagement of interprofessional teams’ (*n* = 6 articles). The least frequently identified determinants (each with one article) were ‘complexity of patient cohort’, ‘advantage of intervention’, ‘intervention framing’, and ‘timeliness of intervention’. Descriptions of the themes within each domain are provided below.

**Table 2:** Implementation determinants of chronic condition hospital avoidance programmes

CFIR domain	CFIR construct	Descriptive inductive themes	Explanation of descriptive themes	Example from text
Domain 1: characteristics of the intervention	Design quality and packaging	Intervention framing	The way in which the intervention is presented to staff	‘One site found that presenting the bundle as best practice resulted in staff being more likely to engage with the project and more willing to complete the bundles.’ [47]
	Complexity/adaptability	Timeliness of intervention	Staff ability to adapt the timing of the intervention for maximum effectiveness	‘Most participants identified timely initiation of NIV as critical to the successful use of this intervention.’ [53]
	Relative advantage	Advantage of intervention	Patients’ perception of the advantage of the intervention versus an alternative	‘Patients felt that the non-face-to-face CCM program provided opportunities for personal health empowerment, including care plans. They acknowledged that care plans were individually designed based on their needs. Patients said their care managers spent long periods of time with them assessing their health needs and goals, and viewed this as a benefit to their health;’ [61]
	Adaptability	Capacity to engage	Factors influencing patients’ ability to fully participate in the intervention	‘One quarter of the individuals approached felt too sick or frail for any activity-related intervention.’ [52]
	Adaptability	Patient interest and perception	Patients’ willingness to participate in the intervention	‘The most common reason for declining participation (cited by four of eleven declining patients [36%]), was lack of interest in being responsible for managing their own insulin therapy given their acute illness.’ [50]
	Adaptability	Psychosocial factors	Issues relating to patients’ psychosocial needs and welfare	‘Social needs of eligible patients are complex and can complicate effective CCM service delivery.’ [61]
	Adaptability	Complexity of clinical conditions influenced by patient complexity	The influence of patients’ medical complexity on the intervention’s effectiveness	‘Complex clinical conditions challenge the effectiveness of CCM programs’ [61]
Domain 2: outer setting	External policy and incentives	External funding	The presence of external financial incentives or other policies regarding reimbursement of organisations and health professionals	‘When the CQUIN was introduced there were financial penalties for non-completion which meant managers were more interested in encouraging staff to complete the bundle’ (Physiotherapist, group 1) [47].
	Cosmopolitanism	Networks with external bodies	The networking of the organisation with others	‘Some hospitals indicated that the networking and examples from other facilities were most helpful, specifically referring to listservs, face-to-face meetings, and webinars. Others indicated that online tools, resources, and access to subject matter experts were most helpful.’ [51].
Domain 3: inner setting	Available resources	Availability of resources	Resources available to the organisation and health professionals, including equipment, training, staffing, and designated time for the intervention	‘Staff highlighted the need for … resourcing and regular training to facilitate QI.’ [55]
	Compatibility	Compatibility and fit	The fit between the intervention and the priorities and existing processes of health professionals and the organisation	‘Projects were often not integrated into the existing health-care field. The care that was offered within telemonitoring did not take into account the provided routine care.’ [58]
	Culture	Engagement of interprofessional team	Relationships between health professionals, including cooperation, communication, and trust	‘Nearly all participants noted the importance of interdisciplinary teamwork…Some participants also cited the importance of teamwork among respiratory therapists … two interviewees indicated that tension with nurses over conflicting priorities could be a barrier to keeping patients on NIV.’ [53]
	Leadership engagement	Leadership influence	The engagement and leadership styles of the organisation leaders	‘Evidence of leadership support is demonstrated by the investment of resources for education, training, and course and conference attendance…. Leaders were actively engaged in and had enduring enthusiasm for both project practices changes, which are currently ongoing.’ [59]
	Compatibility	Alignment with organisation policies, procedures, and systems	Procedural and system design issues impacting health professionals’ ability to implement	‘Several hospital policies were identified as relevant to NIV implementation. The most commonly mentioned policy was not restricting NIV initiation to the ICU… In many cases, participants indicated that they were unfamiliar with or did not know if their hospital had policies related to NIV.’ [53]
	Learning climate	Staff autonomy	The power and ability for health professionals to shape and control their work environment	‘Leadership promoted autonomy by … allowing staff to incorporate EBP, based upon individual unit needs and desires, versus dictating projects and priorities.’ [59]
Domain 4: characteristics of individuals	Knowledge and beliefs about the innovation	Staff buy-in	Health professional engagement with, acceptance and willingness to work with the intervention	‘Nearly all participants highlighted the central role of clinician buy-in with statements indicating that clinicians are generally “on board” with NIV as preferential to intubation.’ [53]
	Self-efficacy	Staff perceptions	Health professional beliefs, motivations, and priorities relating to their work. Closely related to tension for change	‘It was often considered to be outside of the teams’ control and therefore solutions were not considered possible.’ [47]
Domain 5: processes	Champions	Champions	The presence of staff members who are dedicated to promoting and advancing the intervention	‘One team stated that having a champion also allows for the project to be rolled out in new setting more smoothly as it allowed staff to learn from someone they already knew.’ [47]
	Engagement of innovation participants	Engagement with key stakeholders	The involvement of health professional and other stakeholders whose roles are well-positioned to advance the intervention	‘…care teams and patient care were enhanced by the inclusion of care coordinators.’ [61]
	Engagement of innovation participants	Relationships with staff	Patients’ ability to connect and communicate with staff	‘Many of the patients did not feel able to ask healthcare professionals, such as family doctors or specialists, questions about their HF symptoms… Some participants were willing to engage in self-recording their health measurements on a technological device if they were able to develop a connection with healthcare professionals…’ [56]
	Engagement of innovation participants	Patient engagement	The use of strategies by health professionals to attract and involve appropriate patients in the intervention	‘System leaders and health care providers expressed the utility of having the care coordinator on-site to enroll patients in non-face-to-face CCM during their visit to produce greater likelihood of patient acceptance of the co-pay and likelihood to consent to participation.’ [61]
	Reflecting and evaluating	Monitor and feedback	The presence of procedures to monitor and provide feedback to staff about the progress of the intervention	‘Participants at one hospital reported monthly meetings to review patients who failed NIV and attention to the number of days that patients were treated with NIV as an effort to improve NIV use.’ [53]
	Planning	Planning	The degree to which tasks involved for the intervention were developed with staff in advance of implementation	‘Best practices were identified by interviewees who had already implemented non-face-to-face CCM, including staffing models, which patients to enroll in the program, billing practices, and when and how to enroll patients.’ [61]

*EBP* evidence-based practice, *NIV* non-invasive ventilation, *CQUIN* Commissioning for Quality and Innovation framework, *QI* quality improvement, *UPC* unit practice council, *HF* heart failure, *ICU* intensive care unit, *CCM* chronic care management

#### Domain 1: characteristics of the intervention

‘Timeliness of the intervention’ and the importance of patient monitoring were purported to influence successful implementation, where staff ability to ensure early initiation reportedly reduced the likelihood of needing follow-up resource-intensive care [53]. For health professional staff, ‘intervention framing’ as best practice or a more simple way to record existing activities reduced staff concerns regarding performing ‘extra work’ and contributed to their willingness to adopt [47]. The relative ‘advantage of the intervention’ was noted by patients, who felt some interventions offered opportunities for empowerment over their health [61].

Ensuring the appropriateness of the intervention to the local context was reported in several studies. Reduced ‘capacity to engage’ patients was highlighted as a sign of intervention inappropriateness in one study, where patients reported being too sick, busy, or overwhelmed to participate in the intervention, or the travel required was too lengthy [52]. Educational and self-management interventions were also reported as inappropriate to many local settings due to low levels of health literacy, lack of confidence in managing their condition or interacting with health professionals, and making decisions regarding their health [56, 61]. Further, the appropriateness of interventions was, at times, dependent on patients’ access to equipment and technology [60].

Further evidence of the importance of ensuring interventions are appropriate was demonstrated by a lack of ‘patient interest and perception’ in self-management impacting participation in programmes [50, 52, 54, 56]. In some instances, the presence of other co-morbidities requiring more attention, limited patients’ ability to engage [52]. When care was considered useful, comfortable, and applicable to people’s needs, it was accepted more readily [53, 56, 60, 61].

Some interventions were seen as incompatible with patient ‘psychosocial factors’, such as a lack of social support or complex social needs (e.g. substance abuse disorders and depression), which complicated delivery of interventions and impacted some patients’ ability to engage with hospital avoidance programmes [52, 61]. Similarly, the ‘complexity of patient conditions’ were also reported to present a challenge for programmes designed with a more simple or homogenous patient cohort in mind [61].

#### Domain 2: outer setting

‘External funding’ was linked to implementation successes and failures in four of the studies [47, 55, 58, 61]. External financial penalties and incentives encouraged health professionals to complete care bundles and enhance management support for COPD patients [47], as well as facilitate reimbursement of services considered important by system leaders and primary care providers [61]. Pressure on resourcing and staffing lowered care standards for patient follow-up where these incentives were not present [55]. The implementation of telemonitoring was thought to be particularly sensitive to a lack of financial reimbursement, as health professionals then perceived the task to be an extra time-consuming activity on top of clinicians’ existing workload [58].

Building and strengthening ‘networks with external bodies’ through interorganisational coordination was a facilitator for implementation success [59]. However, a lack of access and contact between hospitals and primary care providers prevented successful implementation [51].

#### Domain 3: inner setting

The ‘availability of resources’ to organisations and health professionals, referring to education and training, equipment, staffing, and time commitment, was the most commonly cited inner setting implementation determinant [47, 51, 53–55, 58, 59, 61]. Leadership and managerial support for regular quality improvement training and conference attendance was considered influential to implementation in two included studies [55, 59]. Further, the importance of this support was extended to ensuring adequate amounts of equipment and appropriateness of its quality [53, 58]. In one study, no equipment was provided to staff after the pilot phase of a project, limiting the ability to sustainably scale the innovation beyond the pilot phase [58]. The role of leadership in ensuring an appropriate staffing profile also appeared influential. Concerns were reportedly raised around the adequate number and skill mix of staff, as well as staff turnover affecting implementation [53]. In one instance, the fidelity of the hospital avoidance intervention was attributed, in large part, to staffing issues [54]. Managing additional tasks [54, 58], or the feasibility of dedicating time to different interventions [47, 53, 61], appeared to create issues around competing priorities for health professionals [51].

The importance of ‘alignment with organisation policies, procedures, and systems’ was exemplified by health professionals noting that clarity of procedures and protocols could support implementation success [53, 58]. Often, local systems for managing patients were inappropriate and incompatible with existing health professionals’ workflows, such as incorrect contact details in electronic medical records causing delays and additional work for patient follow-up [54], delayed principal diagnosis coding limiting tracking of admissions [59], and insufficient internal processes for accessing information from other organisations and local conventions that impeded appropriate billing [61].

The included literature suggested that the ‘compatibility and fit’ of hospital avoidance programmes within routine care was a long-term process for health professionals. Time must be taken to learn how to use new tools (e.g. electronic medical records and telemonitoring devices) and incorporate these with existing workflows [58, 61]. Simple adaptations to administrative processes were said to enable implementation success [54, 61], and failure to integrate interventions into existing practice was considered a barrier [53, 58]. Awareness and prioritisation of the programme at different levels of the organisation was also key [51, 59], with examples of health professionals ceasing tasks so they could complete the hospital avoidance intervention [47]. Overlapping of programmes transformed expectations and led to finding support for initiatives from other opinion-leading bodies or individuals [59, 61].

Facilitating communication and buy-in between different hospital departments influenced the ‘engagement of interprofessional teams’ [51, 58]. In situations where communication was open, easy, and receptive, health professionals and other stakeholders were able to build bridges between departments and care settings to enhance teamwork [53, 59, 61]. The importance of interdisciplinary trust in the involvement, delegation, and cooperation amongst colleagues was noted throughout several included studies [51, 58, 61]. Positive relationships that spanned professional boundaries were important for successful implementation; however, tension could arise between professional groups due to conflicting priorities [53]. Sharing tasks amongst different professions also reduced the impact of change on any one group by allowing the workload to be spread [47], which also strengthened continuity of care across team members [61]. The role of hospital leadership in fostering this teamwork supported management of the implementation process [53].

Active and enduring ‘leadership influence’ had a broad impact on how projects and innovations were sustained [59]. Leaders sometimes became more engaged in the presence of external financial incentives [47]. Once engaged, leaders invested in training and education, equipment, and staffing to ensure support [47, 55, 59]. Truly transformational leaders were considered agile and supportive in adapting to challenges without becoming autocratic or cynical.

‘Staff autonomy’, sometimes promoted by effective leadership, reportedly fostered a culture that allowed health professionals to incorporate interventions based on individual needs [59]. A common vision and shared governance arrangements promoted autonomy and ensured the timely delivery of interventions, as it allowed health professionals to independently respond to situations, rather than waiting for instruction [53, 59].

#### Domain 4: characteristics of individuals

‘Staff buy-in’ was demonstrated in three successfully implemented hospital avoidance programmes, where the intervention became the preferred modus operandi [51, 53, 61]. In contexts where there was a lack of staff buy-in, interventions were considered another checklist for health professionals to complete in their already busy day [47].

‘Staff perceptions’ was another characteristic of perceived importance, demonstrated in one study by staff concerns and desires to address variance in practice, creating a tension for change [59]. Higher levels of perceived competence by staff reportedly led to improved patient outcomes [57]. However, solutions were not considered possible in situations where change was perceived as being outside the teams’ personal control [47].

#### Domain 5: process

Health professionals in the included studies noted that ‘champions’ played a critical role in resolving staff engagement issues, as staff showed more willingness to learn from someone they currently work with or are familiar with [47]. Champions were seen to be optimal in another study which had to combat constant staff turnover [59]. ‘Engagement with key stakeholders’ was evident in the close cooperation between a variety of health professionals from different care settings enhancing implementation [58, 59]. In some instances, the inclusion of certain key individuals or roles was considered critical [58, 61].

Promoting ‘patient engagement’ occurred through several activities [60]. Onsite, face-to-face enrolment of patients appeared essential in one study, as emails were often noncompliant [61]. Follow-up phone calls [61] and recruitment of patient family members [58] supported patient engagement and self-management using individualised care plans. Managing processes of ‘monitoring and feedback’ was identified in two studies, where recording patient participation along with outcomes enabled success through regular adaptation [53, 61].

Effective ‘planning’ supported implementation by identifying gaps in the provision of care to facilitate a smoother transition of patients between inpatient and outpatient settings [59]. Clear planning of tasks, such as timing of patient enrolment, staffing models, and billing practices, aided successful enactment of tasks when hospital avoidance interventions were implemented [61].

Many patients were supported by positive ‘relationships with staff’. Feeling connected to one’s health professional increased engagement with self-monitoring [56, 60]. The ease of speaking with someone about conditions, specifically the amount of time that could be spend with health professionals, improved patients’ appreciation of care and perceived importance of the hospital avoidance programme [61].

### Analytical themes: contingent and reciprocal relationships within and between domains for commonly cited implementation determinants

**Fig. 3:**
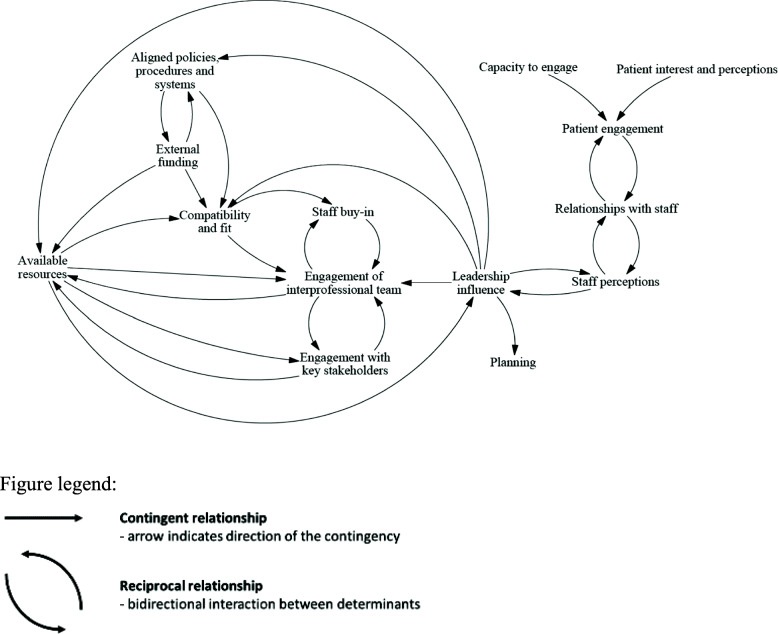
Causal loop diagram for relationships between the most commonly presented determinants across the CFIR domains

We identified both contingent and reciprocal relationships across individual determinants mentioned in three or more studies, categorised according to the CFIR domains (Fig. 3). An example from our coding of the included literature on implementing a new chronic care management reimbursement strategy demonstrates a contingent relationship between the two themes: ‘external funding’ and ‘alignment with organisation, policies, procedures, and systems’. According to our analysis of primary studies, barriers to receiving Medicare or Medicaid reimbursement were *contingent* on system issues preventing the billing of non-face-to-face services when a patient had already been billed by another provider [61]. An example of a reciprocal relationship was identified in our coding between the themes: ‘relationships with staff’ and ‘patient engagement’. According to our analysis, the included literature reported that participants were willing to engage in self-recording health measurements if they developed a connection with their healthcare professionals, the more engaged they were in recording heart failure symptoms the more connected they were to their health professional, and vice versa [56]. It can be seen from these examples how an implementation determinant in one domain could have a large range of interactions with determinants from other domains.

## Discussion

Across a range of contexts, this review found a key set of factors determining the implementation of hospital avoidance programmes for chronic conditions. There were both contingent and reciprocal relationships identified by the reviewers between determinants of implementation across all five CFIR domains, which indicate the importance of considering the entirety of systems, rather than individual components, when embarking upon change processes. We report a causal loop diagram (Fig. 3) that illuminates how strategies to implement hospital avoidance programmes will impact different factors constraining or enabling the desired change.

The frequency of implementation determinant identification within the included studies appeared to be related to the CFIR domain that encapsulates the determinant. A pattern within Fig. 2 appeared to emerge, where inner setting implementation determinants were more frequently identified compared with the characteristics of interventions which were identified less frequently. Determinant frameworks used in implementation science typically include two types of contextual domains: (a) necessary conditions for successful implementation and (b) those more active, driving forces required to achieve desired outcomes [62]. The more frequently mentioned inner setting determinants favour (with some exceptions) active factors, such as ensuring ‘compatibility and fit’, ‘engaging interprofessional teams’, and ‘aligning policies, procedures, and systems’. If the frequency of identification between studies is equated with relative importance, then the integration of intangible, dynamic and active conceptualisations [45, 63] with existing notions of context as concrete and passive would strengthen the design of strategies to facilitate the adoption, implementation, and sustainability of clinical programmes [64].

According to our causal loop diagram, the influence of leadership was an important link between engaging patients and more structural determinants, such as the characteristics of the intervention and inner setting. Previous research has reported on the impact of leadership on engaging staff, colleagues, and patients in decision making, and improving the quality and safety of healthcare [65–67]. However, our system dynamics mapping expands the impact of these relationships by emphasising non-linearities. Leaders can often act as “boundary spanners”, both in their capacity to make resource allocations decisions that may support or hinder implementation, as well as in their position of authority and professional expertise [68]. Therefore, it was somewhat unsurprising that the influence of leadership might link organisational and resourcing factors with patient and staff facing determinants of implementation. The engagement of interprofessional teams acts as another connector between determinants, such as engagement with key stakeholders and staff buy-in, highlighting that efforts to engage key stakeholders and general staff may be flawed without prior engagement of multidisciplinary teams [69]. Stakeholder engagement involves more than obtaining feedback; it also requires the diffusion of decision making power that can affect outcomes, although, there is currently little evidence to indicate best practice stakeholder engagement for improving implementation [70]. Playing a more central role was the compatibility and fit of hospital avoidance programmes with existing workflow and organisational priorities, which is both contingent upon and influential towards several other determinants.

Our causal loop diagram offers a holistic explanation of how factors determining implementation are related, representing a first step towards understanding implementation from a system dynamics perspective. Determinant frameworks in implementation science tend to break down complex phenomena into constituent parts. However, our causal loop diagram (Fig. 3) demonstrates that these determinants are interdependent. For example, the compatibility and fit of chronic condition hospital avoidance programmes is closely related to the availability of resources and external funding. We have identified the specific contextual determinants (i.e. barriers and facilitators) at play for any given implementation effort, so that strategies to address them can be determined, matched, and tailored as part of an implementation planning process [71]. It is important to view factors determining implementation in a holistic manner, rather than breaking them down for the purposes of intervention matching. Applying a reductionist approach in implementation science neglects the possibility that multiple combinations of determinant factors may combine in ways that create unpredictable impacts or negligible effects [62, 72].

In other areas of healthcare, implementation determinants have been identified to inform the design of improvement programmes [29, 30, 73–76]. Tailoring improvement strategies to specifically target barriers and facilitators of change is crucially important [77]; however, mismatching between these important factors has been identified across multiple case studies (e.g. clinician-level strategies employed to address organisational-level barriers) [78]. In complex systems such as healthcare [79–81], influencing large-scale changes in the management of chronic conditions requires us to go beyond simple, linear matching of improvement strategies to implementation determinants [79]. Relationships between implementation determinants have been explored previously to identify potential mechanisms for change strategies [82]. While this is of interest, another important consideration is the associations between determinants from a system dynamics perspective. Better understanding of interactions between components of complex systems and how they give rise to emergent behaviours represents an increasingly valuable framework to apply in implementation science [81]. Explanatory models that highlight the mechanisms by which outcomes are achieved must incorporate all the relevant components of a system (e.g. structure, process, outcomes) and avoid attempts to create an artificially closed system for the purposes of measurement and experiment, which do not represent the real-world environments where implementation occurs.

### Limitations and implications

A limited number of studies met inclusion criteria for our review, highlighting the gap in evidence for the implementation of chronic condition hospital avoidance programmes. Many studies have explored the effectiveness of hospital avoidance programmes and models of care, but few report on the factors enabling or impeding implementation in peer reviewed, English language publications. Previous reviews in either the hospital avoidance [31] or chronic condition [23] literature have returned a similar paucity of high-quality implementation research. Limited retrieval of experimental, quantitative studies means the results from this review were based mostly on non-experimental, qualitative research. Qualitative findings were useful, but without mixed methods triangulation, we are lacking further insight into these findings. The inadequate reporting of ethical approval and the relationships between researchers and participants in our included studies bring into question potential risk of apprehension, ascertainment, and performance bias. Further, weaknesses in reporting of the original study contexts and settings limit the generalisability of findings. As with all systematic reviews, the final dataset is likely to include more successful projects, as unsuccessful attempts are less likely to be published. Ideally, our theorised causal loop diagram for related determinants would be further empirically developed and tested, which is our recommendation for future studies implementing hospital avoidance programmes for people with chronic conditions.

## Conclusion

Value-based care initiatives often involve a focus on hospital avoidance, as part of delivering the right care, to the right person, in the right setting, at the right time. Evidence is still emerging surrounding the different factors that determine how to implement and scale these programmes amongst varying healthcare contexts and settings. The presence of contingent and reciprocal relationships between implementation determinants indicate that efforts to promote practice change require a consideration of whole systems rather than a narrow focus on individual components. High-quality studies are needed to progress from categorisation and descriptions of implementation programmes, towards a more nuanced investigation of implementation processes and mechanisms by which change occurs in complex systems, such as healthcare. Our ongoing research will progress the findings from this review, using empirical data, to further explore the interaction between determinants of hospital avoidance programme implementation, by asking what works, for whom, in which circumstances. The identification of key determinants represents the first step towards developing an optimised, adaptable, evidence-based model for implementing and scaling value-based care initiatives for people with chronic conditions.

## Supplementary information

**Additional file 1.** Search Strategy (title/abstract).

**Additional file 2.** Methodological rigour and risk of bias [48].

## Data Availability

The thematic coding and analysis are available from the corresponding author on reasonable request.

## Abbreviations

AMI: Acute myocardial infarction
CCM: Chronic care management
CFIR: Consolidated Framework for Implementation Research
CHF: Chronic heart failure
COPD: Chronic obstructive pulmonary disease
CQUIN: Commissioning for Quality and Innovation framework
DM: Diabetes mellitus
EBP: Evidence-based practice
HF: Heart failure
ICU: Intensive care unit
NIV: Non-invasive ventilation
PRISMA: Preferred Reporting Items for Systematic Reviews and Meta-analyses
QI: Quality improvement
RCT: Randomised controlled trial
SNF: Skilled nursing facility
UK: United Kingdom
UPC: Unit Practice Council
USA: United States of America
