# Supplementary material for: Avoiding unnecessary hospitalisation for patients with chronic conditions: a systematic review of implementation determinants for hospital avoidance programmes
Source: Implement Sci. 2020 Oct 21;15:91. doi: 10.1186/s13012-020-01049-0 (PMC7579904; doi:10.1186/s13012-020-01049-0)
Supplement: Supplementary file 1 — Additional file 1. Search Strategy (title/abstract). [file 13012_2020_1049_MOESM1_ESM.docx]

## Additional file 1: Search Strategy (title/abstract)

| Strategy | Search string* |
| --- | --- |
|  |  |
| #1 | "Chronic care"  OR  "chronic condition"  OR  "chronic health"  OR  "chronic disease"  OR  "diabetes"  OR  "T2DM"  OR  osteoporo*  OR  "chronic renal disease"  OR  "kidney disease"  OR  "heart failure"  OR  "CHF"  OR  "Chronic obstructive pulmonary disease"  OR  "COPD"  OR  "emphysema”  OR  "COAD"  OR  "arthritis"  OR  "Cardiovascular disease" |
| #2 | “Hospital avoidance” OR readmission* OR “length of stay” OR “length of admission” OR “clinical variation” OR “representation” OR “ED avoidance” OR “emergency department avoidance” |
| #3 | Implement* OR barrier* OR enabler* OR facilitator* |
| #4 | #1 and #2 and #3 |

- MeSH terms used where available
