# Supplementary material for: Avoiding unnecessary hospitalisation for patients with chronic conditions: a systematic review of implementation determinants for hospital avoidance programmes
Source: Implement Sci. 2020 Oct 21;15:91. doi: 10.1186/s13012-020-01049-0 (PMC7579904; doi:10.1186/s13012-020-01049-0)
Supplement: Supplementary file 2 — Additional file 2. Methodological rigour and risk of bias [48]. [file 13012_2020_1049_MOESM2_ESM.docx]

## Appendix 2: Methodological rigour and risk of bias (48)

|  | Study | Abstract and title | Introduction and aims | Method and data | Sampling | Data analysis | Ethics and bias | Findings/ result | Transferability/ generalisability | Implications and usefulness |
| --- | --- | --- | --- | --- | --- | --- | --- | --- | --- | --- |
| 1 | Acton et al (49) | 1 | 2 | 2 | 3 | 3 | 3 | 1 | 3 | 3 |
| 2 | Axon et al (50) | 2 | 2 | 2 | 4 | 2 | 4 | 2 | 3 | 2 |
| 3 | Benzo et al (51) | 2 | 3 | 1 | 1 | 1 | 3 | 1 | 2 | 2 |
| 4 | Fisher et al (52) | 1 | 1 | 1 | 2 | 1 | 2 | 1 | 2 | 2 |
| 5 | Hopkinson et al (53) | 2 | 3 | 3 | 3 | 4 | 4 | 2 | 3 | 4 |
| 6 | Lennox et al (54) | 2 | 1 | 2 | 2 | 2 | 2 | 1 | 2 | 1 |
| 7 | Morton et al (55) | 1 | 2 | 1 | 3 | 3 | 2 | 3 | 3 | 1 |
| 8 | Nguyen et al (56) | 1 | 2 | 1 | 2 | 2 | 1 | 2 | 3 | 1 |
| 9 | Seys et al (57) | 1 | 1 | 2 | 1 | 1 | 2 | 2 | 1 | 2 |
| 10 | Willemse et al (58) | 1 | 1 | 3 | 3 | 3 | 4 | 2 | 3 | 2 |
| 11 | Wood et al (59) | 2 | 1 | 4 | 3 | 4 | 4 | 3 | 3 | 2 |
| 12 | Wright et al (60) | 1 | 3 | 1 | 1 | 2 | 4 | 1 | 2 | 2 |
| 13 | Yeager et al (61) | 1 | 1 | 3 | 1 | 2 | 1 | 1 | 2 | 2 |

*Note.* Good = 1, Fair = 2, Poor = 3, Very poor = 4
